# Genotype Performance Estimation in Targeted Production Environments by Using Sparse Genomic Prediction

**DOI:** 10.3390/plants13213059

**Published:** 2024-10-31

**Authors:** Osval A. Montesinos-López, Paolo Vitale, Guillermo Gerard, Leonardo Crespo-Herrera, Carolina Saint Pierre, Abelardo Montesinos-López, José Crossa

**Affiliations:** 1 Facultad de Telemática, Universidad de Colima, Colima 28040, Colima, Mexico; os78t@gmail.com; 2 International Maize and Wheat Improvement Center (CIMMYT), Km 45, Carretera Mexico-Veracruz, Texcoco de Mora 52640, Edo. de Mexico, Mexico; p.vitale@cgiar.org (P.V.); g.gerard@cgiar.org (G.G.); l.crespo@cgiar.org (L.C.-H.); c.saintpierre@cgiar.org (C.S.P.); 3 Centro Universitario de Ciencias Exactas e Ingenierías (CUCEI), Universidad de Guadalajara, Guadalajara 44430, Jalisco, Mexico; 4 Distinguish Scientist Fellowship Program and Department of Statistics and Operations Research, King Saud University, Riyah 11451, Saudi Arabia; 5 AgCenter, Louisiana State University, Baton Rouge, LA 70803, USA; 6 Colegio de Postgraduados, Montecillos 56230, Edo. de Mexico, Mexico

**Keywords:** genomic prediction, incomplete block designs allocation, sparse testing, random allocation, selection across environments

## Abstract

In plant breeding, Multi-Environment Trials (METs) evaluate candidate genotypes across various conditions, which is financially costly due to extensive field testing. Sparse testing addresses this challenge by evaluating some genotypes in selected environments, allowing for a broader range of environments without significantly increasing costs. This approach integrates genomic information to adjust phenotypic data, leading to more accurate genetic effect estimations. Various sparse testing methods have been explored to optimize resource use. This study employed Incomplete Block Design (IBD) to allocate lines to environments, ensuring not all lines were tested in every environment. We compared IBD to Random line allocation, maintaining a consistent number of environments per line across both methods. The primary objective was to estimate grain yield performance of lines using Genomic Estimated Breeding Values (GEBVs) computed through six Genomic Best Linear Unbiased Predictor (GBLUP) methods. In the first five methods, missing values were predicted before cross-environment adjustment; in the sixth, adjustment was performed directly. Using the Bayesian GBLUP model, we analyzed genotype performance under both IBD and random allocation. Results indicate that computing GEBVs for a target population of environments (TPE) using available phenotype and marker data is effective for selection. The IBD method showed superior performance with less variability compared to random allocation. These findings suggest that using IBD designs can enhance selection accuracy and efficiency, and that pre-adjustment prediction of missing lines may not necessarily improve selection outcomes.

## 1. Introduction

Multi-Environment Trials (MET) are integral to plant breeding, offering critical insights into genotype performance under diverse environmental conditions. These trials assess genotypes across various stresses, including differing climates, soil types, and management practices, with the aim of identifying those that exhibit consistent performance, stability, and adaptability. Breeders must balance selecting genotypes that perform well across a broad spectrum of environments against those tailored to specific conditions. This information is essential for developing varieties suited to regional agricultural needs, ultimately improving crop productivity and resilience. However, MET are costly and resource-intensive due to the extensive field testing involved.

In response to these challenges, sparse testing [1,2,3] combined with genomic prediction presents a transformative approach. Rather than testing every genotype in all environments, sparse testing evaluates subsets of genotypes across specific environments. This allows breeders to expand the number of lines tested without significantly increasing phenotyping costs. Genomic information is then used to adjust phenotypic data across environments, improving the accuracy of genetic effect estimations and compensating for missing phenotypes, thus maintaining high selection accuracy.

The integration of genomic prediction within sparse testing frameworks provides several key advantages. First, it enhances MET efficiency by reducing the dependency on complete phenotypic data, enabling accurate breeding value estimations with fewer trials. This accelerates breeding cycles, reduces costs, and expedites the development of new varieties. Additionally, sparse testing expands the selection intensity by testing more genotypes across diverse environments, increasing the likelihood of identifying superior genotypes. The use of genomic data to predict missing phenotypes allows breeders to focus on high-potential lines, thereby refining the candidate pool and intensifying selection pressure for genetic gain. By strategically distributing genotypes across environments and utilizing genomic predictions, breeders can gain deeper insights into how genotypes respond to environmental variation, ultimately refining breeding strategies and ensuring the development of varieties that are not only high yielding but also resilient to stress.

In plant breeding, evaluating a larger number of lines across more environments is financially costly due to the extensive field testing required [4,5]. Additionally, it is highly challenging to ensure consistent high quality and homogeneous precision across all measurements in every trial. To address this challenge, sparse testing strategies have been proposed [1,2,3]. These strategies involve evaluating some cultivars in certain environments but not in others, enabling testing across a broader range of environments in early-stage yield trials without significantly increasing phenotyping costs. By evaluating only a fraction of lines in each environment, sparse testing effectively increases the number of lines tested across diverse environments and the number of testing environments, thereby allowing for greater selection intensity [2,3,6,7].

When using only phenotypic data to estimate breeding values, the predictions are based solely on observed traits. This method can miss a lot of genetic information, especially for traits with complex inheritance patterns. Traditional models may not fully capture the additive genetic variance because they lack direct genomic information. By incorporating genomic data, the Genomic Estimate Breeding Values (GEBVs) obtained from the Genomic Best Linear Unbiased Predictor (GBLUP) can capture the effects of specific genetic variants (markers) across the genome. This allows the model to more accurately estimate the true genetic potential of every line, even if some environmental factors or random noise influence the observed phenotypes. GEBVs capture a larger proportion of the additive genetic variance by using dense marker data [8], leading to more accurate selection of individuals with desirable genetic traits. Genomic data allows for better predictions across the population, even for individuals that are distantly related or unrelated to those with phenotypic records, because the model is based on genetic similarity. GEBVs allow for earlier and more accurate selection of breeding candidates because the predictions are based on their genetic potential, even before phenotypic traits are fully expressed. Using GEBVs, combined with phenotypic data and genomic markers, provides a more precise and reliable estimate of an individual’s breeding value. This leads to better decision-making in parental selection, improving the efficiency and success of breeding programs.

In sparse testing strategies, one common scenario involves predicting the performance of certain lines that are missing in specific environments while other lines are predicted in others. Montesinos-Lopez et al. [3] and Burgueño et al. [6] addressed the challenge of sparse testing by missing data in multi-environment trials where the idea was to leverage available data from tested lines and environments to predict the performance of untested lines in environments where their data is absent. This method is particularly valuable in plant breeding, where practical constraints often prevent the evaluation of all lines in all environments. However, when evaluating the performance of lines in the TPEs where all lines have been observed at least once across the environments within a specific TPE, the focus shifts. In this situation, the objective is not to predict the performance of unobserved lines in a specific environment, but rather to estimate their overall performance across the TPE. Here, the goal is to adjust the observed phenotypes of the lines by accounting for genomic effects across all environments. By computing the Genomic Estimated Breeding Values (GEBVs) within the previous framework, the impact of genomic factors on phenotypic expression can be quantified, leading to more accurate breeding decisions and enhancing the overall efficiency of the breeding program.

In this paper, we evaluate the estimation capacity of sparse testing methodology using a real data set from South Asian TPEs, which included 25 site/year combinations. This assessment was conducted under the scenario where some lines were considered missing in some environments but present in others. We tested various methods (different structures of the variance-covariance matrix of environmental effects) for estimating the overall performance of the lines across TPEs, including approaches that predict missing values for each line in each environment before performing the overall adjustment, as well as methods where the overall adjustment is performed directly.

## 2. Results

The results are given in four sections. Section 2.1, Section 2.2 and Section 2.3 present the results for data sets TPE_1_2021_2022, TPE_2_2021_2022, and TPE_3_2022_2023, while Section 2.4 provides the results across data sets. Finally, Appendix B and Appendix C provide the results for data sets TPE_1_2022_2023, TPE_2_2022_2023, and TPE_3_2021_2022. The results are provided in terms of Pearson’s correlation (COR), Normalized Root Mean Square Error (NRMSE), and the Percentage of Matching in the top 10% (PM_10) and top 20% (PM_20) of lines for each data set and across data sets. The selection of which data sets results were assigned to the Appendix is random, that is, without any criteria.

### 2.1. TPE_1_2021_2022

**Figure 1:**
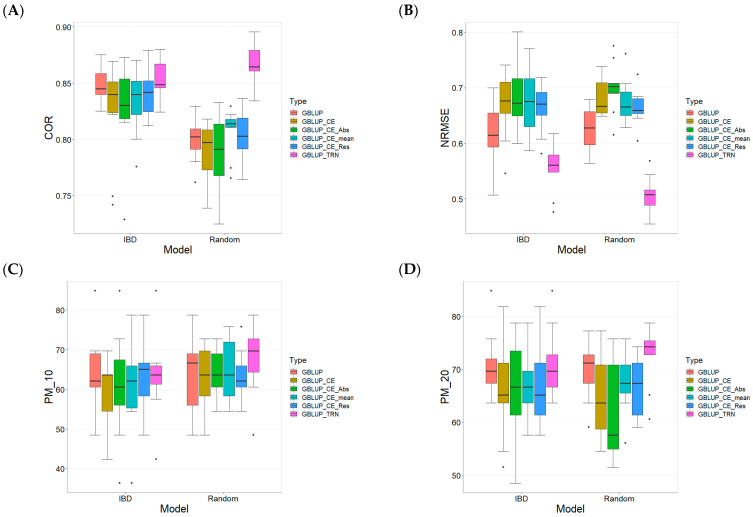
Comparative Performance of genomic prediction models in terms of Pearson´s correlation (COR) (**A**), Normalized Root Mean Square Error (NRMSE) (**B**), Percentage of Matching in top 10% (PM_10) (**C**), and top 20% (PM_20) (**D**) for TPE_1_2021_2022, using Incomplete Block Design Cross-Validation and Random Cross-Validation.

Figure 1 presents the results for the TPE_1_2021_2022 data set under a comparative analysis of the models GBLUP, GBLUP_CE, GBLUP_CE_Abs, GBLUP_CE_mean, GBLUP_CE_Res, and GBLUP_TRN in terms of their predictive efficiency measured by Pearson’s correlation (COR), Normalized Root Mean Square Error (NRMSE), and Percentage of Matching (PM). For more details, see Table A1 in Appendix A.

The Pearson’s correlation computed between observed and predicted values (Figure 1A) shows that the GBLUP_TRN model is the most effective for both the IBD and Random metrics. For the COR metric, the models based on IBD and Random data exhibit different levels of efficiency. In the case of IBD, the GBLUP_TRN model demonstrates the best predictive efficiency with an average of 0.852 and a positive RE compared to the other models, with variation in IBD between 0.323% and 3.188%, with GBLUP_CE being the worst-performing model and GBLUP the second-best. On the other hand, for Random data, GBLUP_TRN also showed the best performance with an average of 0.868 and a positive RE, with variation between 7.422% and 10.547%, with GBLUP_CE_Abs being the worst, showing a higher RE than the training model at 10.547%.

Regarding the NRMSE metric (Figure 1B), the results show that for data based on IBD, the GBLUP_TRN model has the lowest average (0.555). Compared to the other models, it shows an RE ranging from 10.170% to 23.602%, with GBLUP as the best alternative (with an average of 0.612 and an RE of 10.17%) and GBLUP_CE_Abs as the worst model (with an average of 0.686 and an RE of 23.602%). For Random data, GBLUP_TRN also leads with an average of 0.507. The other models have REs between 23.757% and 38.149%, with GBLUP as the best alternative (RE of 23.757%).

The Percentage of Matching “lines/genotypes” (Figure 1C) in the top 10% (PM_10) shows that, for data based on IBD, the GBLUP model has the best predictive efficiency with an average of 65.758% and the GBLUP_TRN model as the second-best with an average of 65.152%. Regarding the Relative Efficiency (RE) of GBLUP_TRN, it is mostly positive except for the conventional model, where they present an RE between −0.922% and 8.586%, with GBLUP_CE as the worst model (with an RE of TRN of 8.586%). For data using Random Cross-Validation, GBLUP_TRN is the best model with an average of 67.879 and a positive RE compared to the other models, which show REs between 5.164% and 8.213%, with GBLUP_CE_mean as the best alternative (RE of 5.164%).

In the PM_20 metric (Figure 1D), for data based on IBD, the GBLUP_TRN model has the best average (70.909%) and an RE compared to the other models ranging from 0.429% to 7.834%, with GBLUP being the best model only after GBLUP_TRN (RE of 0.429%). For Random data, GBLUP_TRN maintains the best predictive efficiency with an average of 72.424%, with an RE ranging from 3.913% to 16.870%. It is important to point out that even the GBLUP_TRN was the best in the four metrics not in all cases was better than the other methods.

### 2.2. TPE_2_2021_2022

**Figure 2:**
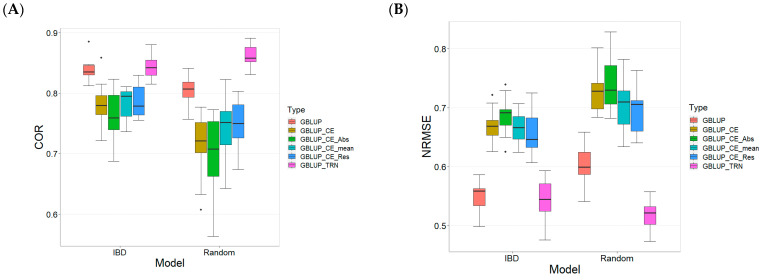

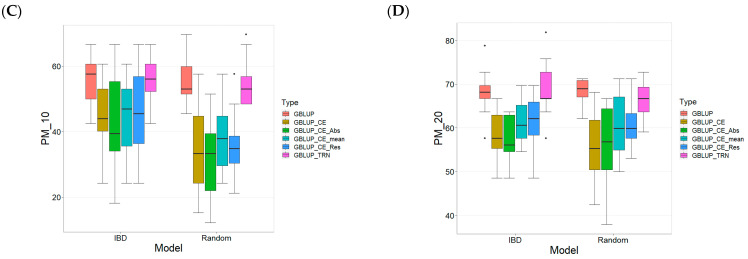
Comparative Performance of genomic prediction models in terms of Pearson´s correlation (COR) (**A**), Normalized Root Mean Square Error (NRMSE) (**B**), Percentage of Matching in top 10% (PM_10) (**C**), and top 20% (PM_20) (**D**) for TPE_2_2021_2022, using Incomplete Block Design Cross-Validation and Random Cross-Validation.

The results for the models evaluated on the TPE_2_2021_2022 data set (Figure 2) also were evaluated with the same metrics: Pearson’s correlation (COR), Normalized Root Mean Square Error (NRMSE), and Percentage of Matching (PM) for the top 10% (PM_10) and top 20% (PM_20). For more details, see Table A2 in Appendix A.

For the Pearson´s correlation metric between observed and predicted values in the testing sets (Figure 2A), the GBLUP_TRN model on IBD data presents the highest mean (0.843) and the low variability (Sd = 0.020). The GBLUP_TRN model on Random data shows a little better performance (mean = 0.862, Sd = 0.019). In contrast, the GBLUP_CE_Abs model applied to Random data has a lower mean (0.700) and higher variability (Sd = 0.065), reflecting greater uncertainty and lower performance. The RE calculated for training relative to the other models (IBD and Random) is positive, ranging from 0.373% to 23.122%, with larger efficiencies obtained using Random CV (from 7.380% to 23.122%).

Regarding the NRMSE metric (Figure 2B), the GBLUP_TRN model on IBD data again stands out with the lowest mean (0.544) and a moderate standard deviation (Sd = 0.035). The GBLUP_CE_Abs model applied to Random data presents the highest mean value (0.738) and a standard deviation of 0.047, suggesting poorer performance in terms of prediction error. The RE of GBLUP_TRN (IBD-Random) ranges from 1.103% to 42.885%.

For the PM_10 metric (Figure 2C), which refers to the Percentage of Matching of the selected top 10% lines under the prediction model with those that are truly the best lines (BLUEs values), the models show significant variations. The GBLUP_TRN model applied to IBD data has the highest mean (56.364) with a standard deviation of 7.719, indicating better performance in selecting the top lines. In contrast, the GBLUP_CE_Abs model applied to Random data has the lowest mean (34.212) with a standard deviation of 13.480, indicating inferior performance in selecting the top lines. The RE of the TRN model is positive in most cases (0.541% to 75.728%), except for the conventional model with Random CV (RE = −1.630%).

In terms of the PM_20 metric (Figure 2D), which refers to the Percentage of Matching of the selected top 20% lines under the prediction model with those that are truly the best lines, the GBLUP_TRN model on IBD data again stands out with a mean of 69.091% and a standard deviation of 6.821. The GBLUP_CE model applied to Random data presents a slightly lower mean (55.758) with a standard deviation of 8.323. As with the previous metric, the RE of the TRN model is positive in most cases (1.333% to 20.00%), except for the conventional model with Random CV (RE = −3.104%).

Overall, the GBLUP_TRN model on IBD data consistently demonstrates better performance in terms of correlation, NRMSE, and Percentage of Matching. The GBLUP_CE model, especially when applied to Random data, tends to show inferior performance and higher variability across all evaluated metrics. However, while GBLUP_TRN performed best across all four metrics, it was not always statistically superior to the other methods.

### 2.3. TPE_3_2022_2023

This section presents the results of the genomic prediction models evaluated on the TPE_3_2022_2023 data, considering the same metrics as before. For more details, see Table A3 in Appendix A.

**Figure 3:**
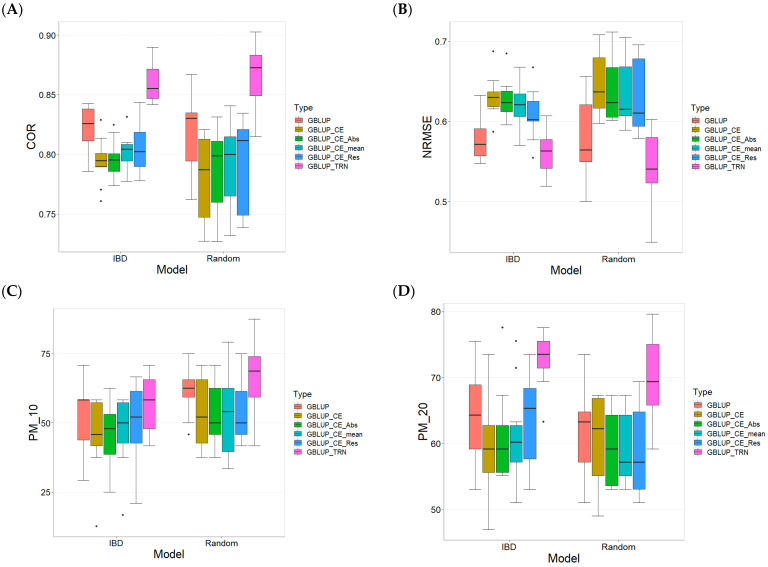
Comparative Performance of genomic prediction models in terms of Pearson´s correlation (COR) (**A**), Normalized Root Mean Square Error (NRMSE) (**B**), Percentage of Matching in top 10% (PM_10) (**C**), and top 20% (PM_20) (**D**) for TPE_3_2022_2023, using Incomplete Block Design Cross-Validation and Random Cross-Validation.

In terms of COR (Figure 3A), the GBLUP_TRN model showed the best performance on IBD data, with a mean of 0.859 and low variability (Sd = 0.016), followed by GBLUP, which also performed well with a mean of 0.822 and even low variability (Sd = 0.019). In comparison, models with Random Cross-Validation showed higher variability, with the GBLUP_TRN model achieving a mean of 0.865 and a standard deviation of 0.026.

For the NRMSE (Figure 3B), the GBLUP_TRN model also led with a mean of 0.561 on IBD and a standard deviation of 0.028, showing higher precision compared to the other models. The second-best GBLUP model had a mean of 0.576 on IBD, with a standard deviation of 0.026. In Random Cross-Validation, the models also showed higher variability, with GBLUP_TRN achieving a mean of 0.539 and a standard deviation of 0.051.

Regarding the Percentage of Matching (Figure 3C) for the top 10% best performance lines, the GBLUP_TRN model with IBD showed outstanding results with a mean of 57.083% and a standard deviation of 11.120. Furthermore, it showed positive REs compared to the other models, which show REs between 7.874% and 25.688%. The second-best GBLUP model achieved a mean of 52.917% on IBD, with a standard deviation of 12.274. In Random Cross-Validation, GBLUP_TRN achieved a mean of 67.083 with a standard deviation of 13.672, showing consistent results with its performance in IBD.

For the Percentage of Matching (Figure 3D) for the top 20% best performance lines, the GBLUP_TRN model on IBD obtained a mean of 72.857 and a standard deviation of 4.308, maintaining its position as the best model. Furthermore, it showed positive REs compared to the other models, which show REs between 12.975% and 22.260%. In Random Cross-Validation, GBLUP_TRN obtained a mean of 69.796 and a standard deviation of 6.857, similar to its performance in IBD.

Overall, the GBLUP_TRN model consistently showed the best performance in terms of COR, NRMSE, and PM in both IBD and Random Cross-Validation, standing out as the most robust and precise model in predicting the best lines. Although GBLUP_TRN showed the best performance across all four metrics, it was not consistently statistically better than the other methods.

### 2.4. Across Data

In this section, the analysis of the results presented across data sets is given under the same model and metrics as before. For more details, see Table A4 in Appendix A.

**Figure 4:**
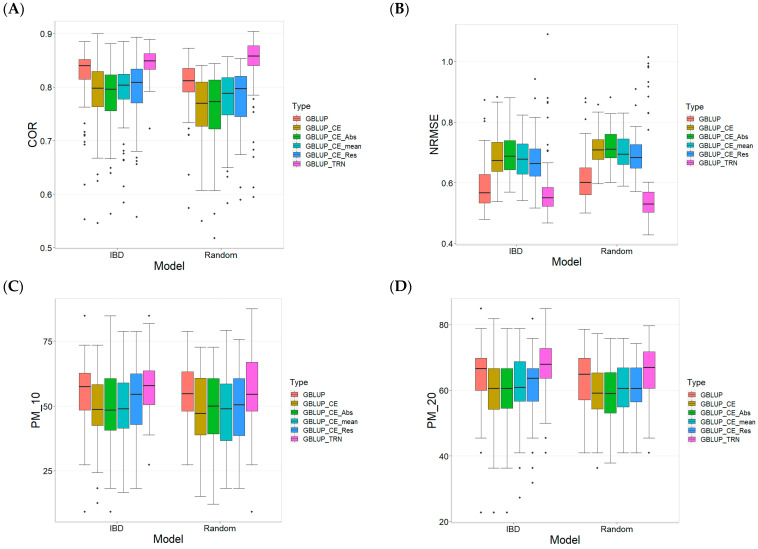
Comparative Performance of genomic prediction models in terms of Pearson´s correlation (COR) (**A**), Normalized Root Mean Square Error (NRMSE) (**B**), Percentage of Matching in top 10% (PM_10) (**C**), and top 20% (PM_20) (**D**) for across data sets using Incomplete Block Design (IBD Cross-Validation and Random Cross-Validation.

In terms of the COR metric (Figure 4A), the GBLUP_TRN model demonstrates the highest predictive efficiency with a mean value of 0.846 for the IBD Cross-Validation method and 0.838 for the Random Cross-Validation method. Other models within the IBD method have REs ranging from 3.234% to 8.353%, while in the Random method, the REs range from 4.538% to 10.748%. This suggests that although other models also have considerable predictive capability, GBLUP_TRN remains the most efficient.

In terms of the NRMSE metric (Figure 4B), GBLUP_TRN also stands out with the lowest mean values (0.584 for IBD and 0.585 for Random), indicating the smallest prediction error and, therefore, the highest prediction accuracy. The REs for other models in IBD range from 2.138% to 19.113%, while in Random, the values fluctuate between 5.335% and 22.390%. This greater range of RE in Random suggests higher variability in the predictive efficiency of the models evaluated under this method.

For the PM_10 metric (Figure 4C), the GBLUP_TRN model shows a mean of 57.311% in IBD and 55.970% in Random, outstanding the other models in both methods. Other models in IBD show an RE between 3.462% and 18.283%, while in Random, the REs range between 2.546% and 15.603%. This implies that, although there are alternative models that can predict effectively, GBLUP_TRN remains the standard in terms of predictive efficiency.

For the PM_20 metric (Figure 4D), GBLUP_TRN has the highest means with 67.715% for IBD and 65.336% for Random, once again surpassing the other models in both methods. The REs for other models in IBD vary between 5.696% and 13.683%, while in Random, the values fluctuate between 3.038% and 10.982%. Again, this indicates that despite the competition, GBLUP_TRN maintains superior predictive efficiency.

Across data, the GBLUP_TRN model consistently stands out as the model with the best predictive capability in all metrics and data types, following almost the same behavior shown in each of the data sets. Other models exhibit variations in their relative efficiency, but none surpass GBLUP_TRN. However, while GBLUP_TRN outperformed across all four metrics, it was not consistently statistically superior to the other methods.

## 3. Discussion

Sparse testing combined with genomic information is gaining significant attention due to its potential to maximize efficiency and reduce costs in plant breeding programs [2,6,7,9]. Traditional methods, which require testing every genotype in all environments, are often prohibitively expensive and time-consuming. Sparse testing, by contrast, allows breeders to strategically evaluate only a subset of genotypes across different environments, thereby expanding the number of environments covered without escalating the costs of field trials. This approach not only preserves the statistical power necessary for accurate predictions but also enhances the ability to model genotype-by-environment interactions, which are crucial for identifying stable and high-performing genotypes. Moreover, in the context of genomic selection, sparse testing leverages advanced computational models to fill in the gaps left by untested genotypes, enabling predictions that are nearly as accurate as those obtained from more exhaustive testing. This combination of cost-effectiveness, improved resource allocation, and robust predictive performance makes sparse testing an attractive strategy for advancing crop improvement efforts.

When sparse testing ensures that each line is evaluated in at least one environment, the use of the genomic information for estimation and selecting the best candidate lines across locations can be computed directly from the measured data. Nonetheless, it is reasonable to assume that incorporating genomic selection methodology could enhance the selection process. By estimating the GEBV for each line, the selection process can utilize both observed and genomic information, potentially leading to more accurate decisions. Therefore, in this paper, we compare the selection process of candidate lines relying solely on GEBV calculated from observed and genomic data

Across the four evaluation metrics—COR, NRMSE, PM_10, and PM_20—the GBLUP_TRN model, which computes GBLUPs using genomic information without employing the prediction methodology, consistently demonstrates superior predictive performance compared to models that incorporate genomic prediction methodology (GBLUP_CE, GBLUP_CE_Abs, GBLUP_CE_mean, and GBLUP_CE_Res). All six methods used genomic information, but only the first five involved prediction. The GBLUP_TRN method adjusted phenotypic values without predicting missing lines in some environments, while the other models used genomic data to predict missing values. For the COR metric, GBLUP_TRN significantly outperforms the other models, with gains ranging from 3.897% to 11.551%. Similarly, in the NRMSE metric, GBLUP_TRN shows notable gains, ranging between 2.229% and 20.628%. Also, across data sets for the PM_10 metric, it leads with gains of 4.467% to 19.582%, and for the PM_20 metric, it excels again with gains ranging from 3.765% to 15.049%. These results collectively highlight GBLUP_TRN as the most efficient and reliable model across all metrics and Cross-Validation methods.

Our best results achieved a COR of 0.844, an NRMSE of 0.596, a PM_10 of 56.692%, and a PM_20 of 67.561% using a model trained with 50% of the data and predicting the remaining 50%. These results are highly promising, as they demonstrate that we can reduce plot costs by 50% while incurring only a modest loss of approximately 15.6% in terms of COR, 43.308% in capturing the top 10% of the best lines, and 32.439% in capturing the top 20% of the best lines.

Although multi-trait prediction models may appear theoretical, they are grounded in substantial empirical research that highlights the utility of shared genetic, environmental, or phenotypic correlations among traits. In our study, the model is applied to real field trial data from a single year, but it involved data collected from 11 different sites, providing a solid foundation for the results. Importantly, the parameter estimation considers both the mean and the variance of the traits, ensuring a comprehensive analysis. It is crucial to underscore that all the data used were derived from field trials, ensuring that the relevance of the multi-trait model is firmly anchored in real-world conditions. This already validates the practical applicability of our approach. Furthermore, the model’s predictions have been tested with actual phenotypic data collected across diverse environments, enhancing confidence in its use for breeding programs.

Among the two Cross-Validation strategies used across the five methods that employed the GS methodology, the IBD Cross-Validation showed slightly better performance. It produced similar mean predicted values but with significantly less variability. This finding is consistent with previous reports by Montesinos-López et al. (2023). The superiority of IBD over the Random allocation of lines can be attributed to its use of a combinatorial algorithm that ensures minimal prediction error during the estimation process. However, the advantage of the IBD method may be limited to smaller data sets, such as those used in this study. Therefore, further research is needed to determine whether this benefit extends to larger data sets.

Finally, our results support the adoption of sparse testing combined with genomic information, as it can significantly reduce costs without substantially sacrificing accuracy. Additionally, the findings for these particular data sets show that when sparse testing guarantees at least one replication per line, the prediction of missing values before the estimation of GEBV across locations does not represent a significant advantage, since the across locations GEBVs can be effectively estimated directly through the Genomic Best Linear Unbiased Predictor (GBLUP) using observed phenotypic and marker data. In such cases, the selection process can be efficiently carried out by directly computing GBLUPs. However, when sparse testing involves many unobserved cultivars across environments, genomic prediction methodology becomes essential to enhance selection accuracy.

## 4. Materials and Methods

### 4.1. Data Sets

The experimental material consisted of 941 wheat new elite lines from CIMMYT, including four checks (NADI, KABILU, NAINA, NINGA) and one local check (Table 1). The genotypes in the data set were evaluated for grain yield (GY) over two crop seasons and across three target populations of environments (TPEs). Of the total genotypes, 444 were tested during the 2021–2022 growing season, while the remaining 497 were evaluated in 2022–2023. In the 2021–2022 season, the genotypes were distributed as follows: 166 in TPE1 (across four locations), 165 in TPE2 (across five locations), and 112 in TPE3 (across two locations). In the 2022–2023 season, 166 genotypes were planted in each of TPE1 (four locations), TPE2 (four locations), and TPE3 (three locations). At each location, the genotypes were planted using an alpha lattice design with two replications. The use of this experimental design with this number of replications had been used for saving costs and for a reasonable parameter estimation, which had provided reasonable results for CIMMYT breeding programs.

### 4.2. Bayesian GBLUP Model

The multi-environment GBLUP model implemented was:

(1) $$Y_{ij}=\mu +L_{i}+g_{j}+gL_{ij}+\epsilon_{ij}$$

where $Y_{ij}$ is the BLUE of each *i*th line at every *j*th environment, μ is the grand mean, $L_{i},\;i=1,\ldots ,I,$ are the Random effects of locations, distributed as $L={\left(L_{1},\ldots ,L_{I}\right)}^{T}∼N_{J}\left(0,\sigma_{E}^{2}{Ω}_{E}\right)$, where ${Ω}_{E}$ denotes the covariance relationship matrix of environments, and $\sigma_{E}^{2}$ denotes the variance component of environments. In addition, $g_{j},$ j=1,…,J, are the Random effects of lines, $gL_{ij}$ are the Random effects of location-line interaction, and $\epsilon_{ij}$ are Random error components in the model assumed to be independent normal Random variables with mean 0 and variance $\sigma^{2}$. Furthermore, it is assumed that $g={\left(g_{1},\ldots ,g_{J}\right)}^{T}∼N_{J}\left(0,\sigma_{g}^{2}G\right)$, where G is the genomic relationship-matrix [10], and $\sigma_{g}^{2}$ denotes the genetic variance component; $gL={\left(gL_{11},\ldots ,gL_{1J},\ldots ,\;gL_{IJ}\right)}^{T}∼N_{IJ}\left(0,\sigma_{gL}^{2}\left(Z_{g}GZ_{g}^{T}{}^{\circ}Z_{E}{Ω}_{E}Z_{E}^{T}\right)\right)$, $Z_{g}$ denotes the incidence matrix for the vector of additive genetic effects, $\sigma_{gL}^{2}$ denotes the variance component of the genotype by environment interaction and ° denotes the Hadamard product, and $Z_{E}\;$ represents the incidence matrix for the effects of environments (i.e., the matrix that connects the phenotypes with environments). The implementation of this model was done in the BGLR library [11]. Finally, $\epsilon_{ij}$ corresponds to the residual error assuming $\epsilon_{ij}∼N_{J}\left(0,\sigma_{\epsilon }^{2}\right),$ where $\sigma_{\epsilon }^{2}$ is the error variance.

Therefore, with small differences in the Equation (1) we end up implementing six methods, which are the following:

GBLUP: this method uses an identity matrix for the covariance relationship matrix between environments, that is, ${Ω}_{E}=I_{I}$.

GBLUP_CE: this method uses an unstructured covariance relationship matrix between environments, but this was estimated in a first stage using also the BGLR package but with the Multitrait() function. More explicitly, the unstructured covariance matrix was implemented with the following R code:

$$\begin{array}{l} \mathrm{fmUN}<-\mathrm{Multitrait}(y=Y,\mathrm{ETA}=\mathrm{list}(\mathrm{Lines}=\mathrm{list}(K=G,\mathrm{model}=“\mathrm{RKHS}”,\mathrm{Cov}=\mathrm{list}(\mathrm{type}=“\mathrm{UN}”,\mathrm{df}0=5,\\ S0=\mathrm{diag}(\mathrm{No}\_\mathrm{Env}))),\mathrm{nIter}=20000,\mathrm{burnIn}=10000,\mathrm{verbose}=\mathrm{FALSE}), \end{array}$$

In this fitting process, the Y matrix contained in each column the information of each environment. Also, those positions in the testing set in the Y matrix were filled with missing values, “NA”. **G** denotes the genomic relationship matrix computed as explained before, RKHS denotes the reproducing kernel Hilber spaces model, in type was specified “UN,” which denotes that it was implemented an unstructured covariance matrix, df0 denotes a hyperameter that denotes the prior degrees of freedom, S0 denotes a prior scale matrix, this is a diagonal matrix of dimension the number of environments (No_Env), nIter denotes the number of iterations used in the training process, and burnIn denotes the number of iterations that are discarded for estimation of the parameters. After this first training process, the genetic and residual covariance matrices of environments were extracted as:

Cov_Env = fmUN$ETA$Lines$Cov$Omega

Res_Cov_Env = fmUN$resCov$R

Finally, this GBLUP_CE method was implemented as the GBLUP method but using in ${Ω}_{E}=Cov\_Env$.

GBLUP_CE_Abs: this method uses an unstructured covariance relationship matrix with absolute values for the genetic covariances between environments. That is, this was implemented as the GBLUP method but using in ${Ω}_{E}=abs({\mathrm{Cov}}_{\mathrm{Env}})$, whre abs denotes the absolute values of each component of the Cov_Env matrix.

GBLUP_CE_mean: this method uses an unstructured covariance relationship matrix for the genetic covariance between environments. This is composed as the addition of the genetic and residual covariances divided by 2. That is, this method was implemented as the GBLUP method but using ${Ω}_{E}=0.5*{Cov}_{Env}+0.5*Res\_Cov\_Env$.

GBLUP_CE_Res: this method uses an unstructured covariance relationship matrix for the genetic covariance between environments, and it is composed of a residual covariance. That is, this method was implemented as the GBLUP method but using ${Ω}_{E}=Res\_Cov\_Env$.

GBLUP_TRN: this method uses the repeated lines in some environments to compute the genomic estimate breeding value (GEBV) of the cultivars by adjusting the phenotype of the observed cultivars using genomic information. For this reason, we did not apply the prediction methodology in this approach. Instead, we computed the GEBVs using the observed data, and the model is provided in Equation (1).

When using the above six methods, it is useful to define: (1) **GBLUP**: linear mixed model where the relationship matrix is the genomic relationship matrix, which is derived from molecular markers (Single Nucleotide Polymorphisms, SNPs). This allows for the inclusion of genomic information in the estimation of breeding values; (2) **GEBV**: The outcome of the GBLUP model is the GEBV, which represents the estimated breeding value of an individual based on its genetic makeup, as inferred from the marker data. These GEBVs represent the genetic potential of individuals, helping breeders make more informed selection decisions.

It is important to note that all six methods utilized genomic information, but only the first five employed a prediction methodology. The last method, GBLUP_TRN, used genomic information but did not predict missing values for lines in certain environments. Instead, GBLUP_TRN adjusted the phenotypic values using genomic data without specifically predicting missing values. In contrast, the other models used genomic information to predict each missing value individually. Also, it is important to point out that model (1) was implemented for each year separately. For this reason, we are not making any assumptions about the similarity of years.

### 4.3. Allocation of Lines to Environments

Under both allocation methods, balanced incomplete block design (IBD) and Random, we use the following notation: *J* represents the number of lines (treatments), *k* represents the environment (location or block) size, *I* represents the number of environments, and *r* represents the number of replicates of line *j* in the entire design. In IBD, *k* will be less than *J*, meaning you cannot assign all treatments in each environment. Ensuring an equal number of replications is crucial for minimizing variance in pairwise comparisons. Thus, with $r_{i}=r$ for all treatments, the total number of observations in the experiment *N* is given by: N = J⋅r = b⋅k.

#### 4.3.1. Allocation Under a Balanced Incomplete Block Design (IBD)

A balanced incomplete block design (IBD) is one where all pairs of treatments occur together within a block an equal number of times (*λ*). Specifically, $\lambda_{jj}$ denotes the number of times treatment *j* occurs with j´ in an environment (block). To generate this sparse allocation of lines to environments, we can use the function find.BIB() from the R package crossdes.

For example, Montesinos-López et al. [3] assume that we have *J* =12 treatments and *I* = 4 environments, and we decide to use *N*_*TRN* = 36 (75%) of the total individuals in the training set (TRN_set). The number of lines per environment can be obtained by solving (*k*I = N_TRN) for *k*, which results in *k* = N_TRN/I. This gives us *k* = 36/4 = 9 treatments per block. The corresponding elements for the training set can then be obtained with the function find.BIB(12, 4, 9) from the crossdes package in R. The numbers used in the function find.BIB() represent the treatments, the environments (blocks), and the lines per environment, respectively. Finally, the lines that will be tested in the field (TRN set) are shown in Table 2.

According to Table 2, each treatment is present in three blocks and missing in one block. It is important to note that all the lines shown in Table 2 correspond to the training set, while those not allocated in each environment constitute the testing set. For example, in environment 1, the testing set includes treatments L2, L8, and L10; in environment 2, the testing set comprises treatments L4, L6, and L12; in environment 3, the testing set consists of treatments L1, L7, and L9; and in environment 4, the testing set includes treatments L3, L5, and L11. It is also important to point out that the find.BIB() function does not always guarantee a BIB design. When a full BIB design is not possible, it only guarantees a partially BIB design.

#### 4.3.2. Random Allocation (Random) of Lines to Environments

According to Montesinos-López et al. [3], starting from a balanced data set with *J* genotypes (lines) and *I* environments (locations), the Random allocation of lines to environments was performed so that each line is repeated in approximately *r* out of *I* environments, and all environments are of the same size (*k*). The algorithm for this Random allocation is as follows:

Step 1. Compute $=\left\lceil \frac{J\times r}{I}\right\rceil$ (the least integer greater than or equal to $\frac{J\times r}{I}$). Then, randomly allocate *k* out of *J* lines to the first environment.

Step 2. Repeat this process for the second environment by randomly allocating *k* out of *J* lines.

Step 3. Continue this process for each environment up to the *I*th environment, with the restriction that lines allocated to a particular environment are present in fewer than *r* environments, ideally in exactly *r* environments. Lines that do not satisfy this restriction are not candidates for allocation to that environment.

### 4.4. Cross-Validation Strategy

To evaluate the predictive performance, we used Cross-Validation with 10 Random partitions. In each partition, 50% of the data was used for training and 50% for testing but assuming that each line was observed in at least one environment. This means that, for example, each particular line should be observed in two environments and missed in the other two, assuming that the data set under evaluation contains four environments. This type of cross validation belongs to simulating tested lines in tested environments, as described with details in [12]. Then, using the observed and predicted values in each testing set, the Normalized Root Mean Square Error (NRMSE), Pearson’s correlation (COR), and the Percentage of Matching in the top 10% (PM_10) and top 20% (PM_20) of lines across the 10 random partitions were computed. It is important to point out that in the testing set the metrics of each random partition were computed, and what is reported as prediction performance is the average of the 10 random partitions for each data set. These metrics were used to assess prediction performance in each data set under study. While focusing solely on COR may simplify the presentation, the use of four evaluation criteria was intentional to provide a comprehensive assessment of the model’s performance. Each metric offers a unique perspective, allowing for a more robust and nuanced understanding of the results. Relying on just one criterion, such as COR, could overlook important aspects of model behavior that other metrics, like NRMSE or PM_10/PM_20, help capture. Thus, we believe that presenting multiple evaluation criteria adds depth and value to the analysis, rather than unnecessary complexity.

For comparing the prediction performance between the GBLUP_TRN method and the remaining methods in terms of COR, PM_10, and PM_20, we computed the relative efficiency as:

$$RE=\frac{Average\;performance\;of\;the\;GBLUP\_TRN\;method}{Average\;performance\;of\;any\;of\;the\;other\;five\;methods}\times 100$$

where RE denotes the relative efficiency of the GBLUP_TRN method with respect to any other of the five methods. If the value of RE is greater than 100, then the GBLUP_TRN method is better than the other method in terms of COR, PM_10, or PM_20. While if the RE is less than 100, the GBLUP_TRN method is less efficient (with more prediction error) than the other method.

While in terms of NRMSE, the RE was computed as

$$RE=\frac{Average\;performance\;of\;any\;of\;the\;other\;five\;methods}{Average\;performance\;of\;the\;GBLUP\_TRN\;method}\times 100$$

Again, if the value of RE is greater than 100, then the GBLUP_TRN method is better than the other method in terms of NRMSE. While if the RE is less than 100, the GBLUP_TRN method is less efficient (with more prediction error) than the other method in terms of NRMSE.

It is important to point out that the use of the RE for comparison methods was used under both types of Cross-Validation, IBD and Random.

### 4.5. Genotypic Data

The genotypic data set consisted of approximately 18,000 SNP markers, generated using the Genotyping-by-Sequencing (GBS) technique. The genotyping was performed on an Illumina HiSeq2500 sequencer at Kansas State University. Stringent quality control was carried out using TASSEL v5.0 software (https://tassel.bitbucket.io, 10 June 2023). During the initial data curation, markers with a minor allele frequency (MAF) below 5% were filtered out, and those with more than 50% missing data were excluded. The remaining missing genotypes were imputed using samples from the marginal distribution of marker genotypes, that is, $x_{ij}\sim$ Bernoulli $\left(p_{j}\right),$ where $p_{j}$ is the estimated allele frequency computed from the non-missing genotypes.

## 5. Conclusions

Our research supports the idea that sparse testing can maximize efficiency and reduce costs in plant breeding programs. In sparse testing, we try to predict the genetic merit of each line based on available data. In this scenario, where only a subset of lines is tested in specific environments, the goal is to estimate the genetic performance of all genotypes across all environments, rather than simply predicting missing values for those genotypes not available in specific environments. This estimation allows breeders to adjust the observed data, accounting for genetic effects captured by markers, and to make informed decisions based on these adjusted phenotypes.

We have shown that the use of sparse testing combined with genomic information is an efficient approach for selecting candidate lines for the next generation. In these cases, candidate lines can be selected simply by computing the GBLUPs using the observed data. Nonetheless, we recognize that there are several alternatives for allocating lines to environments where many lines may not be present in all environments. In such scenarios, the use of genomic prediction methodology becomes crucial. Therefore, we encourage further research to design novel approaches to sparse testing that can enhance the efficiency of plant breeding programs.

## Figures and Tables

**Table 1:** Description of the wheat data sets. MAF denotes the Minor Allele Frequency, and PMV denotes the threshold of Percentage of Missing Values.

No.	Data	Lines	Markers	Env	MAF	PMV
1	TPE_1_2021_2022	166	18238	4	0.05	50%
2	TPE_1_2022_2023	166	18238	6	0.05	50%
3	TPE_2_2021_2022	166	18238	4	0.05	50%
4	TPE_2_2022_2023	165	18238	6	0.05	50%
5	TPE_3_2021_2022	112	18238	2	0.05	50%
6	TPE_3_2022_2023	166	18238	3	0.05	50%

**Table 2:** Allocation of J = 12 lines to I = 4 environments under a BIB design. This allocation represents the training set (75%), the size of the environment is equal to 9, and each line is repeated r = b(k)/J = 36/12 = three times.

Environments	1	2	3	4	5	6	7	8	9
Env1	L1	L3	L4	L5	L6	L7	L9	L11	L12
Env2	L1	L2	L3	L5	L7	L8	L9	L10	L11
Env3	L2	L3	L4	L5	L6	L8	L10	L11	L12
Env4	L1	L2	L4	L6	L7	L8	L9	L10	L12

**Figure A1:**
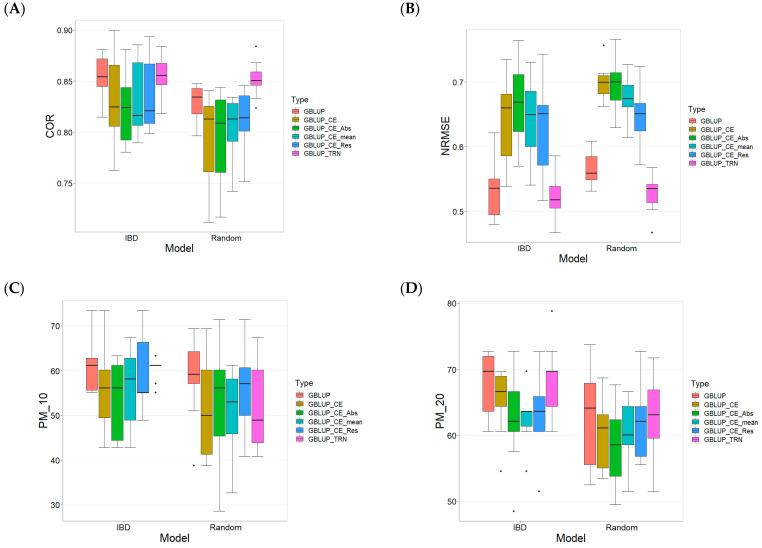
Comparative Performance of genomic prediction models in terms of Pearson’s correlation (COR) (**A**), Normalized Root Mean Square Error (NRMSE) (**B**), Percentage of Matching in top 10% (PM_10) (**C**), and top 20% (PM_20) (**D**) for the **TPE_1_2022_2023** data set under Incomplete Block Design (IBD Cross-Validation and Random Cross-Validation.

**Figure A2:**
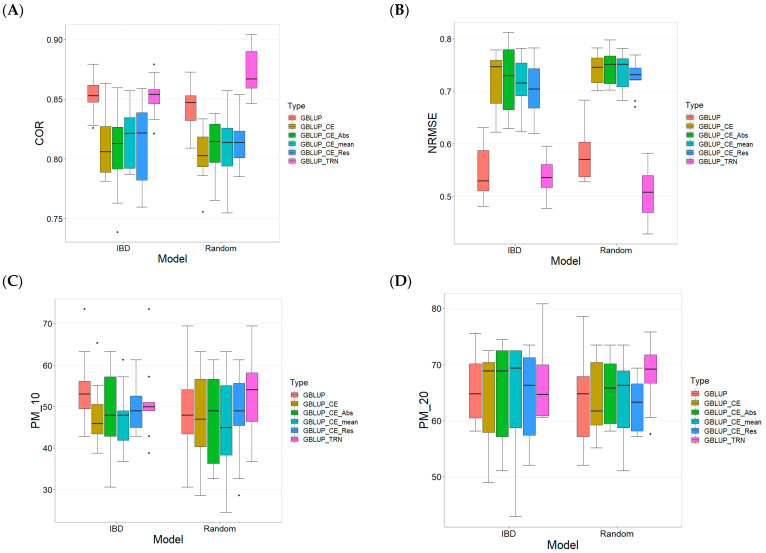
Comparative Performance of genomic prediction models in terms of Pearson´s correlation (COR) (**A**), Normalized Root Mean Square Error (NRMSE) (**B**), Percentage of Matching in top 10% (PM_10) (**C**), and top 20% (PM_20) (**D**) for the **TPE_2_2022_2023** data set under Incomplete Block Design (IBD Cross-Validation and Random Cross-Validation.

**Figure A3:**
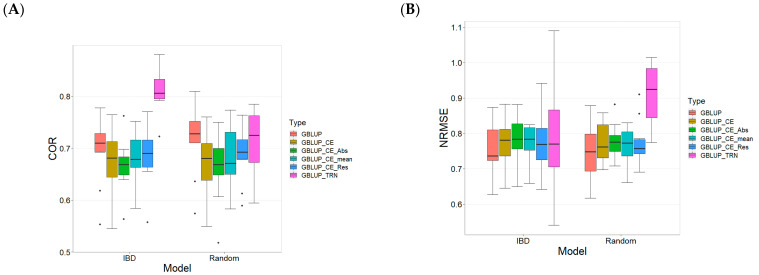

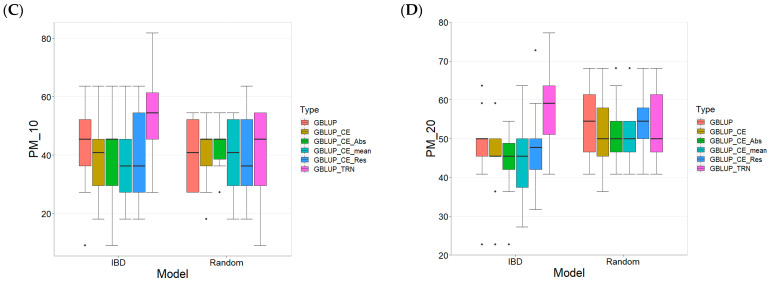
Comparative Performance of genomic prediction models in terms of Pearson´s correlation (COR) (**A**), Normalized Root Mean Square Error (NRMSE) (**B**), Percentage of Matching in top 10% (PM_10) (**C**), and top 20% (PM_20) (**D**) for the **TPE_3_2021_2022** data set under Incomplete Block Design (IBD Cross-Validation and Random Cross-Validation.

## Data Availability

The phenotypic and marker data used in this study can be downloaded from the following link: https://github.com/osval78/Sparse_testing_Across, 10 September 2024.

## Appendix A

**Table A1:** Comparative Performance of genomic prediction models in terms of Pearson´s correlation (COR) (A), Normalized Root Mean Square Error (NRMSE) (B), Percentage of Matching in top 10% (PM_10) (C), and top 20% (PM_20) (D) for the **TPE_1_2021_2022** data set under Incomplete Block Design (IBD Cross-Validation and Random Cross-Validation.

METRIC	CV	MODEL	MIN	MEAN	MEDIAN	MAX	SD	RE (%)
COR	IBD	GBLUP	0.825	0.849	0.845	0.875	0.017	0.323
COR	IBD	GBLUP_CE	0.742	0.825	0.840	0.869	0.044	3.188
COR	IBD	GBLUP_CE_Abs	0.729	0.829	0.830	0.873	0.041	2.759
COR	IBD	GBLUP_CE_mean	0.776	0.833	0.840	0.870	0.029	2.176
COR	IBD	GBLUP_CE_Res	0.812	0.842	0.842	0.879	0.022	1.085
COR	IBD	GBLUP_TRN	0.824	0.852	0.849	0.880	0.019	0.000
COR	Random	GBLUP	0.762	0.799	0.802	0.829	0.019	8.592
COR	Random	GBLUP_CE	0.739	0.789	0.797	0.818	0.025	9.898
COR	Random	GBLUP_CE_Abs	0.725	0.785	0.791	0.833	0.037	10.547
COR	Random	GBLUP_CE_mean	0.766	0.808	0.814	0.829	0.021	7.422
COR	Random	GBLUP_CE_Res	0.764	0.804	0.803	0.837	0.021	7.971
COR	Random	GBLUP_TRN	0.834	0.868	0.864	0.896	0.017	0.000
NRMSE	IBD	GBLUP	0.507	0.612	0.615	0.700	0.062	10.170
NRMSE	IBD	GBLUP_CE	0.546	0.670	0.676	0.741	0.060	20.681
NRMSE	IBD	GBLUP_CE_Abs	0.600	0.686	0.672	0.801	0.057	23.602
NRMSE	IBD	GBLUP_CE_mean	0.587	0.675	0.675	0.771	0.057	21.525
NRMSE	IBD	GBLUP_CE_Res	0.582	0.664	0.671	0.719	0.043	19.667
NRMSE	IBD	GBLUP_TRN	0.476	0.555	0.561	0.618	0.043	0.000
NRMSE	Random	GBLUP	0.564	0.627	0.628	0.679	0.038	23.757
NRMSE	Random	GBLUP_CE	0.649	0.681	0.667	0.738	0.034	34.315
NRMSE	Random	GBLUP_CE_Abs	0.615	0.700	0.702	0.776	0.045	38.149
NRMSE	Random	GBLUP_CE_mean	0.628	0.675	0.666	0.761	0.038	33.242
NRMSE	Random	GBLUP_CE_Res	0.604	0.664	0.659	0.724	0.031	31.017
NRMSE	Random	GBLUP_TRN	0.455	0.507	0.508	0.568	0.033	0.000
PM_10	IBD	GBLUP	48.485	65.758	62.121	84.848	11.521	−0.922
PM_10	IBD	GBLUP_CE	42.424	60.000	63.636	69.697	9.236	8.586
PM_10	IBD	GBLUP_CE_Abs	36.364	60.909	60.606	84.848	13.282	6.965
PM_10	IBD	GBLUP_CE_mean	36.364	60.303	62.121	78.788	11.014	8.040
PM_10	IBD	GBLUP_CE_Res	48.485	63.333	65.152	78.788	8.505	2.871
PM_10	IBD	GBLUP_TRN	42.424	65.152	63.636	84.848	12.392	0.000
PM_10	Random	GBLUP	48.485	63.333	66.667	78.788	9.416	7.177
PM_10	Random	GBLUP_CE	48.485	62.727	63.636	72.727	9.040	8.213
PM_10	Random	GBLUP_CE_Abs	54.545	63.939	63.636	72.727	5.794	6.161
PM_10	Random	GBLUP_CE_mean	54.545	64.545	63.636	75.758	7.831	5.164
PM_10	Random	GBLUP_CE_Res	54.545	63.333	62.121	75.758	6.136	7.177
PM_10	Random	GBLUP_TRN	48.485	67.879	69.697	78.788	8.713	0.000
PM_20	IBD	GBLUP	63.636	70.606	69.697	84.848	6.235	0.429
PM_20	IBD	GBLUP_CE	51.515	65.758	65.152	81.818	8.812	7.834
PM_20	IBD	GBLUP_CE_Abs	48.485	66.667	66.667	78.788	9.367	6.364
PM_20	IBD	GBLUP_CE_mean	57.576	66.970	66.667	78.788	6.136	5.882
PM_20	IBD	GBLUP_CE_Res	57.576	66.970	65.152	81.818	7.619	5.882
PM_20	IBD	GBLUP_TRN	63.636	70.909	69.697	84.848	6.731	0.000
PM_20	Random	GBLUP	59.091	69.697	71.212	77.273	5.249	3.913
PM_20	Random	GBLUP_CE	54.545	65.000	63.636	77.273	7.871	11.422
PM_20	Random	GBLUP_CE_Abs	51.515	61.970	57.576	75.758	9.270	16.870
PM_20	Random	GBLUP_CE_mean	56.061	67.576	67.424	75.758	5.449	7.175
PM_20	Random	GBLUP_CE_Res	59.091	66.818	67.424	74.242	5.553	8.390
PM_20	Random	GBLUP_TRN	60.606	72.424	74.242	78.788	5.430	0.000

**Table A2:** Comparative Performance of genomic prediction models in terms of Pearson´s correlation (COR) (A), Normalized Root Mean Square Error (NRMSE) (B), Percentage of Matching in top 10% (PM_10) (C), and top 20% (PM_20) (D) for the TPE_2_2021_2022 data set under Incomplete Block Design (IBD Cross-Validation and Random Cross-Validation.

METRIC	CV	MODEL	MIN	MEAN	MEDIAN	MAX	SD	RE (%)
COR	IBD	GBLUP	0.812	0.839	0.835	0.885	0.019	0.373
COR	IBD	GBLUP_CE	0.722	0.780	0.780	0.858	0.040	8.067
COR	IBD	GBLUP_CE_Abs	0.687	0.763	0.759	0.823	0.044	10.373
COR	IBD	GBLUP_CE_mean	0.736	0.784	0.795	0.811	0.026	7.502
COR	IBD	GBLUP_CE_Res	0.755	0.787	0.778	0.829	0.027	7.094
COR	IBD	GBLUP_TRN	0.815	0.843	0.842	0.880	0.020	0.000
COR	Random	GBLUP	0.757	0.803	0.807	0.841	0.025	7.380
COR	Random	GBLUP_CE	0.607	0.714	0.721	0.777	0.056	20.780
COR	Random	GBLUP_CE_Abs	0.563	0.700	0.708	0.773	0.065	23.122
COR	Random	GBLUP_CE_mean	0.642	0.743	0.752	0.823	0.049	16.086
COR	Random	GBLUP_CE_Res	0.674	0.749	0.750	0.803	0.040	15.099
COR	Random	GBLUP_TRN	0.830	0.862	0.858	0.891	0.019	0.000
NRMSE	IBD	GBLUP	0.499	0.550	0.559	0.586	0.025	1.103
NRMSE	IBD	GBLUP_CE	0.626	0.670	0.669	0.721	0.028	23.294
NRMSE	IBD	GBLUP_CE_Abs	0.625	0.687	0.692	0.739	0.034	26.414
NRMSE	IBD	GBLUP_CE_mean	0.624	0.667	0.666	0.707	0.027	22.592
NRMSE	IBD	GBLUP_CE_Res	0.607	0.658	0.647	0.725	0.037	20.943
NRMSE	IBD	GBLUP_TRN	0.476	0.544	0.545	0.593	0.035	0.000
NRMSE	Random	GBLUP	0.541	0.604	0.599	0.659	0.035	16.982
NRMSE	Random	GBLUP_CE	0.684	0.729	0.728	0.801	0.040	41.120
NRMSE	Random	GBLUP_CE_Abs	0.682	0.738	0.730	0.828	0.047	42.885
NRMSE	Random	GBLUP_CE_mean	0.634	0.705	0.710	0.782	0.044	36.489
NRMSE	Random	GBLUP_CE_Res	0.640	0.695	0.706	0.763	0.039	34.585
NRMSE	Random	GBLUP_TRN	0.473	0.517	0.522	0.558	0.027	0.000
PM_10	IBD	GBLUP	42.424	56.061	57.576	66.667	7.593	0.541
PM_10	IBD	GBLUP_CE	24.242	44.848	43.939	60.606	11.935	25.676
PM_10	IBD	GBLUP_CE_Abs	18.182	42.424	39.394	66.667	15.843	32.857
PM_10	IBD	GBLUP_CE_mean	24.242	44.545	46.970	60.606	12.041	26.531
PM_10	IBD	GBLUP_CE_Res	24.242	45.758	45.455	66.667	14.246	23.179
PM_10	IBD	GBLUP_TRN	42.424	56.364	56.061	66.667	7.719	0.000
PM_10	Random	GBLUP	45.455	55.758	53.030	69.697	7.850	−1.630
PM_10	Random	GBLUP_CE	15.152	34.545	33.333	57.576	14.441	58.772
PM_10	Random	GBLUP_CE_Abs	12.121	31.212	33.333	51.515	13.480	75.728
PM_10	Random	GBLUP_CE_mean	24.242	38.182	37.879	57.576	10.222	43.651
PM_10	Random	GBLUP_CE_Res	21.212	35.758	34.848	57.576	10.860	53.390
PM_10	Random	GBLUP_TRN	48.485	54.848	53.030	69.697	7.752	0.000
PM_20	IBD	GBLUP	57.576	68.182	68.182	78.788	5.578	1.333
PM_20	IBD	GBLUP_CE	48.485	58.485	57.576	66.667	6.069	18.135
PM_20	IBD	GBLUP_CE_Abs	48.485	57.576	56.061	63.636	5.151	20.000
PM_20	IBD	GBLUP_CE_mean	54.545	61.515	60.606	69.697	5.354	12.315
PM_20	IBD	GBLUP_CE_Res	48.485	61.818	62.121	69.697	6.420	11.765
PM_20	IBD	GBLUP_TRN	57.576	69.091	66.667	81.818	6.821	0.000
PM_20	Random	GBLUP	62.121	68.333	68.939	71.212	2.984	−3.104
PM_20	Random	GBLUP_CE	42.424	55.758	55.303	68.182	8.323	18.750
PM_20	Random	GBLUP_CE_Abs	37.879	55.909	56.818	66.667	9.753	18.428
PM_20	Random	GBLUP_CE_mean	50.000	60.606	59.848	71.212	7.035	9.250
PM_20	Random	GBLUP_CE_Res	53.030	60.909	59.848	71.212	5.569	8.706
PM_20	Random	GBLUP_TRN	59.091	66.212	66.667	72.727	4.520	0.000

**Table A3:** Comparative Performance of genomic prediction models in terms of Pearson´s correlation (COR) (A), Normalized Root Mean Square Error (NRMSE) (B), Percentage of Matching in top 10% (PM_10) (C), and top 20% (PM_20) (D) for the TPE_3_2022_2023 data set under Incomplete Block Design (IBD Cross-Validation and Random Cross-Validation.

METRIC	CV	MODEL	MIN	MEAN	MEDIAN	MAX	SD	RE(%)
COR	IBD	GBLUP	0.786	0.822	0.826	0.843	0.019	4.499
COR	IBD	GBLUP_CE	0.761	0.794	0.795	0.829	0.019	8.185
COR	IBD	GBLUP_CE_Abs	0.774	0.796	0.795	0.825	0.016	7.923
COR	IBD	GBLUP_CE_mean	0.777	0.801	0.804	0.831	0.016	7.260
COR	IBD	GBLUP_CE_Res	0.778	0.806	0.802	0.844	0.021	6.640
COR	IBD	GBLUP_TRN	0.842	0.859	0.855	0.890	0.016	0.000
COR	Random	GBLUP	0.762	0.821	0.830	0.867	0.034	5.468
COR	Random	GBLUP_CE	0.727	0.779	0.787	0.821	0.037	11.032
COR	Random	GBLUP_CE_Abs	0.727	0.788	0.799	0.831	0.035	9.795
COR	Random	GBLUP_CE_mean	0.732	0.790	0.800	0.841	0.036	9.563
COR	Random	GBLUP_CE_Res	0.739	0.792	0.812	0.835	0.040	9.274
COR	Random	GBLUP_TRN	0.815	0.865	0.873	0.903	0.026	0.000
NRMSE	IBD	GBLUP	0.547	0.576	0.571	0.633	0.026	2.696
NRMSE	IBD	GBLUP_CE	0.587	0.631	0.630	0.687	0.026	12.611
NRMSE	IBD	GBLUP_CE_Abs	0.595	0.628	0.623	0.685	0.025	11.961
NRMSE	IBD	GBLUP_CE_mean	0.570	0.619	0.621	0.668	0.027	10.478
NRMSE	IBD	GBLUP_CE_Res	0.554	0.608	0.602	0.667	0.031	8.463
NRMSE	IBD	GBLUP_TRN	0.519	0.561	0.563	0.607	0.028	0.000
NRMSE	Random	GBLUP	0.500	0.576	0.564	0.656	0.054	6.813
NRMSE	Random	GBLUP_CE	0.597	0.646	0.637	0.708	0.041	19.827
NRMSE	Random	GBLUP_CE_Abs	0.601	0.638	0.624	0.712	0.040	18.238
NRMSE	Random	GBLUP_CE_mean	0.589	0.633	0.615	0.705	0.041	17.446
NRMSE	Random	GBLUP_CE_Res	0.579	0.631	0.611	0.696	0.046	17.015
NRMSE	Random	GBLUP_TRN	0.449	0.539	0.540	0.602	0.051	0.000
PM_10	IBD	GBLUP	29.167	52.917	58.333	70.833	12.274	7.874
PM_10	IBD	GBLUP_CE	12.500	45.417	45.833	58.333	14.089	25.688
PM_10	IBD	GBLUP_CE_Abs	25.000	46.250	47.917	62.500	11.189	23.423
PM_10	IBD	GBLUP_CE_mean	16.667	47.083	50.000	58.333	12.888	21.239
PM_10	IBD	GBLUP_CE_Res	20.833	50.000	52.083	66.667	14.027	14.167
PM_10	IBD	GBLUP_TRN	41.667	57.083	58.333	70.833	11.120	0.000
PM_10	Random	GBLUP	45.833	61.250	62.500	75.000	8.345	9.524
PM_10	Random	GBLUP_CE	37.500	53.333	52.083	70.833	12.699	25.781
PM_10	Random	GBLUP_CE_Abs	37.500	53.750	50.000	70.833	11.015	24.806
PM_10	Random	GBLUP_CE_mean	33.333	52.917	54.167	79.167	15.472	26.772
PM_10	Random	GBLUP_CE_Res	41.667	53.750	50.000	75.000	11.360	24.806
PM_10	Random	GBLUP_TRN	41.667	67.083	68.750	87.500	13.672	0.000
PM_20	IBD	GBLUP	53.061	64.490	64.286	75.510	7.212	12.975
PM_20	IBD	GBLUP_CE	46.939	59.592	59.184	73.469	7.440	22.260
PM_20	IBD	GBLUP_CE_Abs	55.102	61.020	59.184	77.551	7.034	19.398
PM_20	IBD	GBLUP_CE_mean	51.020	61.224	60.204	75.510	7.390	19.000
PM_20	IBD	GBLUP_CE_Res	53.061	63.469	65.306	73.469	7.034	14.791
PM_20	IBD	GBLUP_TRN	63.265	72.857	73.469	77.551	4.308	0.000
PM_20	Random	GBLUP	51.020	62.245	63.265	73.469	6.887	12.131
PM_20	Random	GBLUP_CE	48.980	60.204	62.245	67.347	7.215	15.932
PM_20	Random	GBLUP_CE_Abs	53.061	59.184	59.184	67.347	5.610	17.931
PM_20	Random	GBLUP_CE_mean	53.061	59.184	57.143	67.347	5.181	17.931
PM_20	Random	GBLUP_CE_Res	51.020	58.776	57.143	69.388	6.991	18.750
PM_20	Random	GBLUP_TRN	59.184	69.796	69.388	79.592	6.857	0.000

**Table A4:** Comparative Performance of genomic prediction models in terms of Pearson´s correlation (COR) (A), Normalized Root Mean Square Error (NRMSE) (B), Percentage of Matching in top 10% (PM_10) (C), and top 20% (PM_20) (D) for the Across-data sets under Incomplete Block Design (IBD Cross-Validation and Random Cross-Validation.

METRIC	CV	MODEL	MIN	MEAN	MEDIAN	MAX	SD	RE (%)
COR	IBD	GBLUP	0.553	0.819	0.840	0.885	0.064	3.234
COR	IBD	GBLUP_CE	0.545	0.786	0.798	0.900	0.066	7.625
COR	IBD	GBLUP_CE_Abs	0.563	0.781	0.796	0.881	0.067	8.353
COR	IBD	GBLUP_CE_Res	0.557	0.795	0.809	0.894	0.062	6.341
COR	IBD	GBLUP_CE_mean	0.584	0.791	0.804	0.886	0.062	6.906
COR	IBD	GBLUP_TRN	0.723	0.846	0.849	0.890	0.028	0.000
COR	Random	GBLUP	0.574	0.802	0.812	0.873	0.054	4.538
COR	Random	GBLUP_CE	0.549	0.758	0.770	0.841	0.064	10.548
COR	Random	GBLUP_CE_Abs	0.517	0.757	0.773	0.844	0.072	10.748
COR	Random	GBLUP_CE_Res	0.589	0.777	0.798	0.854	0.058	7.893
COR	Random	GBLUP_CE_mean	0.583	0.773	0.788	0.857	0.061	8.470
COR	Random	GBLUP_TRN	0.594	0.838	0.858	0.904	0.067	0.000
NRMSE	IBD	GBLUP	0.480	0.597	0.567	0.873	0.093	2.138
NRMSE	IBD	GBLUP_CE	0.538	0.685	0.674	0.882	0.072	17.235
NRMSE	IBD	GBLUP_CE_Abs	0.570	0.696	0.687	0.881	0.071	19.113
NRMSE	IBD	GBLUP_CE_Res	0.517	0.674	0.664	0.942	0.079	15.271
NRMSE	IBD	GBLUP_CE_mean	0.541	0.681	0.678	0.824	0.068	16.618
NRMSE	IBD	GBLUP_TRN	0.467	0.584	0.551	1.090	0.113	0.000
NRMSE	Random	GBLUP	0.500	0.616	0.601	0.879	0.080	5.335
NRMSE	Random	GBLUP_CE	0.597	0.711	0.708	0.858	0.056	21.566
NRMSE	Random	GBLUP_CE_Abs	0.601	0.716	0.711	0.882	0.061	22.390
NRMSE	Random	GBLUP_CE_Res	0.572	0.689	0.684	0.909	0.065	17.804
NRMSE	Random	GBLUP_CE_mean	0.589	0.698	0.695	0.830	0.059	19.245
NRMSE	Random	GBLUP_TRN	0.428	0.585	0.530	1.014	0.156	0.000
PM_10	IBD	GBLUP	9.091	55.393	57.576	84.848	12.836	3.462
PM_10	IBD	GBLUP_CE	12.500	48.956	48.732	73.469	12.735	17.067
PM_10	IBD	GBLUP_CE_Abs	9.091	48.453	48.485	84.848	14.124	18.283
PM_10	IBD	GBLUP_CE_Res	18.182	51.463	54.545	78.788	13.780	11.365
PM_10	IBD	GBLUP_CE_mean	16.667	48.828	48.980	78.788	13.503	17.373
PM_10	IBD	GBLUP_TRN	27.273	57.311	57.955	84.848	10.870	0.000
PM_10	Random	GBLUP	27.273	54.581	54.824	78.788	12.279	2.546
PM_10	Random	GBLUP_CE	15.152	48.416	47.159	72.727	14.479	15.603
PM_10	Random	GBLUP_CE_Abs	12.121	48.571	50.000	72.727	14.630	15.234
PM_10	Random	GBLUP_CE_Res	18.182	49.453	50.510	75.758	13.908	13.179
PM_10	Random	GBLUP_CE_mean	18.182	48.628	48.980	79.167	14.235	15.100
PM_10	Random	GBLUP_TRN	9.091	55.970	54.545	87.500	14.294	0.000
PM_20	IBD	GBLUP	22.727	64.066	66.667	84.848	10.220	5.696
PM_20	IBD	GBLUP_CE	22.727	59.846	60.606	81.818	10.276	13.149
PM_20	IBD	GBLUP_CE_Abs	22.727	59.565	60.606	78.788	10.733	13.683
PM_20	IBD	GBLUP_CE_Res	31.818	61.310	63.636	81.818	9.749	10.448
PM_20	IBD	GBLUP_CE_mean	27.273	60.405	60.915	78.788	10.281	12.102
PM_20	IBD	GBLUP_TRN	40.909	67.715	67.929	84.848	8.298	0.000
PM_20	Random	GBLUP	40.909	63.410	64.899	78.571	8.349	3.038
PM_20	Random	GBLUP_CE	36.364	59.309	59.184	77.273	8.738	10.163
PM_20	Random	GBLUP_CE_Abs	37.879	58.871	59.091	75.758	8.495	10.982
PM_20	Random	GBLUP_CE_Res	40.909	60.861	60.606	74.242	7.140	7.353
PM_20	Random	GBLUP_CE_mean	40.909	60.746	60.606	75.758	7.820	7.557
PM_20	Random	GBLUP_TRN	40.909	65.336	67.007	79.592	8.849	0.000

## Appendix C

**Table A5:** Comparative performance of genomic prediction models in terms of Pearson´s correlation (COR) (A), Normalized Root Mean Square Error (NRMSE) (B), Percentage of Matching in top 10% (PM_10) (c), and top 20% (PM_20) (D) for TPE_1_2022_2023, TPE_2_2022_2023, and TPE_3_2021_2022 data sets under Incomplete Block Design (IBD Cross-Validation and Random Cross-Validation.

DATA TPE	METRIC	CV	MODEL	MIN	MEAN	MEDIAN	MAX	SD	RE (%)
1_2022_2023	COR	IBD	GBLUP	0.815	0.856	0.854	0.881	0.020	0.090
1_2022_2023	COR	IBD	GBLUP_CE	0.763	0.831	0.825	0.900	0.042	3.053
1_2022_2023	COR	IBD	GBLUP_CE_Abs	0.780	0.824	0.824	0.881	0.035	3.937
1_2022_2023	COR	IBD	GBLUP_CE_mean	0.789	0.834	0.816	0.886	0.037	2.729
1_2022_2023	COR	IBD	GBLUP_CE_Res	0.799	0.836	0.821	0.894	0.036	2.457
1_2022_2023	COR	IBD	GBLUP_TRN	0.818	0.856	0.856	0.884	0.019	0.000
1_2022_2023	COR	Random	GBLUP	0.796	0.829	0.835	0.848	0.019	2.808
1_2022_2023	COR	Random	GBLUP_CE	0.711	0.793	0.813	0.841	0.044	7.475
1_2022_2023	COR	Random	GBLUP_CE_Abs	0.717	0.796	0.809	0.844	0.046	6.971
1_2022_2023	COR	Random	GBLUP_CE_mean	0.742	0.804	0.813	0.834	0.031	5.929
1_2022_2023	COR	Random	GBLUP_CE_Res	0.751	0.814	0.814	0.846	0.029	4.641
1_2022_2023	COR	Random	GBLUP_TRN	0.823	0.852	0.851	0.884	0.017	0.000
1_2022_2023	NRMSE	IBD	GBLUP	0.480	0.534	0.536	0.622	0.047	1.620
1_2022_2023	NRMSE	IBD	GBLUP_CE	0.538	0.641	0.660	0.735	0.069	22.029
1_2022_2023	NRMSE	IBD	GBLUP_CE_Abs	0.570	0.668	0.669	0.764	0.061	27.138
1_2022_2023	NRMSE	IBD	GBLUP_CE_mean	0.541	0.642	0.650	0.730	0.062	22.126
1_2022_2023	NRMSE	IBD	GBLUP_CE_Res	0.517	0.629	0.651	0.743	0.071	19.691
1_2022_2023	NRMSE	IBD	GBLUP_TRN	0.467	0.526	0.518	0.586	0.036	0.000
1_2022_2023	NRMSE	Random	GBLUP	0.531	0.566	0.559	0.608	0.027	7.270
1_2022_2023	NRMSE	Random	GBLUP_CE	0.662	0.698	0.699	0.756	0.027	32.271
1_2022_2023	NRMSE	Random	GBLUP_CE_Abs	0.629	0.699	0.700	0.766	0.040	32.349
1_2022_2023	NRMSE	Random	GBLUP_CE_mean	0.614	0.674	0.675	0.727	0.038	27.727
1_2022_2023	NRMSE	Random	GBLUP_CE_Res	0.572	0.646	0.651	0.724	0.042	22.348
1_2022_2023	NRMSE	Random	GBLUP_TRN	0.467	0.528	0.535	0.568	0.029	0.000
1_2022_2023	PM_10	IBD	GBLUP	55.102	61.020	61.224	73.469	5.965	−1.003
1_2022_2023	PM_10	IBD	GBLUP_CE	42.857	55.306	56.122	73.469	9.448	9.225
1_2022_2023	PM_10	IBD	GBLUP_CE_Abs	42.857	53.673	56.122	63.265	8.499	12.548
1_2022_2023	PM_10	IBD	GBLUP_CE_mean	42.857	55.714	58.163	67.347	8.714	8.425
1_2022_2023	PM_10	IBD	GBLUP_CE_Res	48.980	59.184	55.102	73.469	8.106	2.069
1_2022_2023	PM_10	IBD	GBLUP_TRN	55.102	60.408	61.224	63.265	2.395	0.000
1_2022_2023	PM_10	Random	GBLUP	38.776	58.367	59.184	69.388	8.616	−10.490
1_2022_2023	PM_10	Random	GBLUP_CE	38.776	51.020	50.000	69.388	11.095	2.400
1_2022_2023	PM_10	Random	GBLUP_CE_Abs	28.571	52.857	56.122	71.429	12.707	−1.158
1_2022_2023	PM_10	Random	GBLUP_CE_mean	32.653	51.020	53.061	61.224	8.870	2.400
1_2022_2023	PM_10	Random	GBLUP_CE_Res	40.816	55.918	57.143	71.429	8.507	−6.569
1_2022_2023	PM_10	Random	GBLUP_TRN	40.816	52.245	48.980	67.347	10.371	0.000
1_2022_2023	PM_20	IBD	GBLUP	60.606	67.576	69.697	72.727	4.959	1.345
1_2022_2023	PM_20	IBD	GBLUP_CE	54.545	65.455	66.667	69.697	4.781	4.630
1_2022_2023	PM_20	IBD	GBLUP_CE_Abs	48.485	62.424	62.121	72.727	6.577	9.709
1_2022_2023	PM_20	IBD	GBLUP_CE_mean	54.545	63.333	63.636	69.697	4.391	8.134
1_2022_2023	PM_20	IBD	GBLUP_CE_Res	51.515	63.030	63.636	72.727	5.496	8.654
1_2022_2023	PM_20	IBD	GBLUP_TRN	60.606	68.485	69.697	78.788	5.190	0.000
1_2022_2023	PM_20	Random	GBLUP	52.525	62.727	64.141	73.737	7.399	−0.644
1_2022_2023	PM_20	Random	GBLUP_CE	53.535	59.899	61.111	68.687	5.174	4.047
1_2022_2023	PM_20	Random	GBLUP_CE_Abs	49.495	58.485	58.586	67.677	6.032	6.563
1_2022_2023	PM_20	Random	GBLUP_CE_mean	51.515	60.404	60.101	66.667	4.898	3.177
1_2022_2023	PM_20	Random	GBLUP_CE_Res	55.556	61.818	62.121	72.727	5.710	0.817
1_2022_2023	PM_20	Random	GBLUP_TRN	51.515	62.323	63.131	71.717	6.442	0.000
2_2022_2023	COR	IBD	GBLUP	0.826	0.853	0.853	0.879	0.017	−0.054
2_2022_2023	COR	IBD	GBLUP_CE	0.781	0.811	0.806	0.863	0.026	5.137
2_2022_2023	COR	IBD	GBLUP_CE_Abs	0.739	0.806	0.813	0.859	0.035	5.770
2_2022_2023	COR	IBD	GBLUP_CE_mean	0.787	0.817	0.821	0.857	0.025	4.361
2_2022_2023	COR	IBD	GBLUP_CE_Res	0.760	0.813	0.822	0.859	0.035	4.796
2_2022_2023	COR	IBD	GBLUP_TRN	0.821	0.852	0.854	0.879	0.017	0.000
2_2022_2023	COR	Random	GBLUP	0.809	0.844	0.847	0.873	0.020	3.416
2_2022_2023	COR	Random	GBLUP_CE	0.756	0.803	0.803	0.833	0.023	8.682
2_2022_2023	COR	Random	GBLUP_CE_Abs	0.766	0.810	0.814	0.838	0.023	7.748
2_2022_2023	COR	Random	GBLUP_CE_mean	0.755	0.812	0.814	0.857	0.030	7.541
2_2022_2023	COR	Random	GBLUP_CE_Res	0.785	0.816	0.814	0.854	0.023	7.041
2_2022_2023	COR	Random	GBLUP_TRN	0.847	0.873	0.867	0.904	0.020	0.000
2_2022_2023	NRMSE	IBD	GBLUP	0.481	0.547	0.529	0.631	0.053	2.124
2_2022_2023	NRMSE	IBD	GBLUP_CE	0.623	0.720	0.747	0.779	0.055	34.472
2_2022_2023	NRMSE	IBD	GBLUP_CE_Abs	0.629	0.724	0.730	0.813	0.064	35.132
2_2022_2023	NRMSE	IBD	GBLUP_CE_mean	0.623	0.713	0.716	0.782	0.051	33.050
2_2022_2023	NRMSE	IBD	GBLUP_CE_Res	0.620	0.702	0.704	0.783	0.054	31.088
2_2022_2023	NRMSE	IBD	GBLUP_TRN	0.476	0.536	0.535	0.595	0.038	0.000
2_2022_2023	NRMSE	Random	GBLUP	0.528	0.578	0.570	0.683	0.050	13.901
2_2022_2023	NRMSE	Random	GBLUP_CE	0.702	0.741	0.746	0.783	0.028	46.186
2_2022_2023	NRMSE	Random	GBLUP_CE_Abs	0.703	0.745	0.751	0.798	0.033	46.998
2_2022_2023	NRMSE	Random	GBLUP_CE_mean	0.682	0.737	0.752	0.782	0.034	45.376
2_2022_2023	NRMSE	Random	GBLUP_CE_Res	0.670	0.728	0.732	0.769	0.031	43.464
2_2022_2023	NRMSE	Random	GBLUP_TRN	0.428	0.507	0.508	0.582	0.051	0.000
2_2022_2023	PM_10	IBD	GBLUP	42.857	53.878	53.061	73.469	9.187	−4.924
2_2022_2023	PM_10	IBD	GBLUP_CE	38.776	48.163	45.918	65.306	7.587	6.356
2_2022_2023	PM_10	IBD	GBLUP_CE_Abs	30.612	48.367	47.959	63.265	10.758	5.907
2_2022_2023	PM_10	IBD	GBLUP_CE_mean	36.735	47.143	47.959	61.224	7.959	8.658
2_2022_2023	PM_10	IBD	GBLUP_CE_Res	42.857	49.592	48.980	61.224	6.164	3.292
2_2022_2023	PM_10	IBD	GBLUP_TRN	38.776	51.224	50.000	73.469	9.250	0.000
2_2022_2023	PM_10	Random	GBLUP	30.612	48.776	47.959	69.388	10.861	8.368
2_2022_2023	PM_10	Random	GBLUP_CE	28.571	47.959	46.939	63.265	11.312	10.213
2_2022_2023	PM_10	Random	GBLUP_CE_Abs	32.653	46.939	48.980	61.224	11.137	12.609
2_2022_2023	PM_10	Random	GBLUP_CE_mean	24.490	45.102	44.898	63.265	12.224	17.195
2_2022_2023	PM_10	Random	GBLUP_CE_Res	28.571	47.959	48.980	61.224	10.680	10.213
2_2022_2023	PM_10	Random	GBLUP_TRN	36.735	52.857	54.082	69.388	9.546	0.000
2_2022_2023	PM_20	IBD	GBLUP	58.163	65.816	64.796	75.510	6.108	1.445
2_2022_2023	PM_20	IBD	GBLUP_CE	48.980	63.878	68.878	72.449	8.807	4.524
2_2022_2023	PM_20	IBD	GBLUP_CE_Abs	51.020	65.612	68.878	74.490	9.064	1.761
2_2022_2023	PM_20	IBD	GBLUP_CE_mean	42.857	64.388	69.388	72.449	10.209	3.696
2_2022_2023	PM_20	IBD	GBLUP_CE_Res	52.041	64.388	66.327	73.469	7.983	3.696
2_2022_2023	PM_20	IBD	GBLUP_TRN	60.606	66.768	64.646	80.808	7.086	0.000
2_2022_2023	PM_20	Random	GBLUP	52.041	63.367	64.796	78.571	8.296	7.438
2_2022_2023	PM_20	Random	GBLUP_CE	55.102	64.082	61.735	73.469	6.679	6.241
2_2022_2023	PM_20	Random	GBLUP_CE_Abs	58.163	65.408	65.816	73.469	6.131	4.086
2_2022_2023	PM_20	Random	GBLUP_CE_mean	51.020	63.980	66.327	73.469	7.038	6.410
2_2022_2023	PM_20	Random	GBLUP_CE_Res	57.143	62.755	63.265	69.388	4.935	8.486
2_2022_2023	PM_20	Random	GBLUP_TRN	57.576	68.081	69.192	75.758	5.537	0.000
3_2021_2022	COR	IBD	GBLUP	0.553	0.697	0.710	0.777	0.067	16.619
3_2021_2022	COR	IBD	GBLUP_CE	0.545	0.674	0.681	0.765	0.061	20.506
3_2021_2022	COR	IBD	GBLUP_CE_Abs	0.563	0.666	0.668	0.762	0.050	22.112
3_2021_2022	COR	IBD	GBLUP_CE_mean	0.584	0.678	0.678	0.752	0.052	19.807
3_2021_2022	COR	IBD	GBLUP_CE_Res	0.557	0.688	0.690	0.771	0.060	18.106
3_2021_2022	COR	IBD	GBLUP_TRN	0.723	0.813	0.806	0.880	0.044	0.000
3_2021_2022	COR	Random	GBLUP	0.574	0.716	0.728	0.809	0.067	−0.909
3_2021_2022	COR	Random	GBLUP_CE	0.549	0.671	0.681	0.760	0.061	5.734
3_2021_2022	COR	Random	GBLUP_CE_Abs	0.517	0.662	0.669	0.750	0.065	7.245
3_2021_2022	COR	Random	GBLUP_CE_mean	0.583	0.681	0.671	0.774	0.060	4.249
3_2021_2022	COR	Random	GBLUP_CE_Res	0.589	0.687	0.692	0.764	0.054	3.223
3_2021_2022	COR	Random	GBLUP_TRN	0.594	0.710	0.725	0.785	0.069	0.000
3_2021_2022	NRMSE	IBD	GBLUP	0.627	0.763	0.736	0.873	0.077	−2.867
3_2021_2022	NRMSE	IBD	GBLUP_CE	0.645	0.777	0.781	0.882	0.069	−1.053
3_2021_2022	NRMSE	IBD	GBLUP_CE_Abs	0.650	0.783	0.783	0.881	0.066	−0.300
3_2021_2022	NRMSE	IBD	GBLUP_CE_mean	0.659	0.774	0.783	0.824	0.052	−1.490
3_2021_2022	NRMSE	IBD	GBLUP_CE_Res	0.642	0.780	0.768	0.942	0.086	−0.644
3_2021_2022	NRMSE	IBD	GBLUP_TRN	0.540	0.785	0.770	1.090	0.151	0.000
3_2021_2022	NRMSE	Random	GBLUP	0.617	0.747	0.748	0.879	0.087	−18.209
3_2021_2022	NRMSE	Random	GBLUP_CE	0.697	0.773	0.762	0.858	0.055	−15.380
3_2021_2022	NRMSE	Random	GBLUP_CE_Abs	0.708	0.778	0.776	0.882	0.050	−14.896
3_2021_2022	NRMSE	Random	GBLUP_CE_mean	0.661	0.762	0.773	0.830	0.054	−16.607
3_2021_2022	NRMSE	Random	GBLUP_CE_Res	0.691	0.773	0.757	0.909	0.065	−15.412
3_2021_2022	NRMSE	Random	GBLUP_TRN	0.774	0.914	0.924	1.014	0.082	0.000
3_2021_2022	PM_10	IBD	GBLUP	9.091	42.727	45.455	63.636	16.625	25.532
3_2021_2022	PM_10	IBD	GBLUP_CE	18.182	40.000	40.909	63.636	13.687	34.091
3_2021_2022	PM_10	IBD	GBLUP_CE_Abs	9.091	39.091	45.455	63.636	14.876	37.209
3_2021_2022	PM_10	IBD	GBLUP_CE_mean	18.182	38.182	36.364	63.636	16.487	40.476
3_2021_2022	PM_10	IBD	GBLUP_CE_Res	18.182	40.909	36.364	63.636	16.735	31.111
3_2021_2022	PM_10	IBD	GBLUP_TRN	27.273	53.636	54.545	81.818	14.501	0.000
3_2021_2022	PM_10	Random	GBLUP	27.273	40.000	40.909	54.545	12.272	2.273
3_2021_2022	PM_10	Random	GBLUP_CE	18.182	40.909	45.455	54.545	11.539	0.000
3_2021_2022	PM_10	Random	GBLUP_CE_Abs	27.273	42.727	45.455	54.545	9.630	−4.255
3_2021_2022	PM_10	Random	GBLUP_CE_mean	18.182	40.000	40.909	54.545	12.999	2.273
3_2021_2022	PM_10	Random	GBLUP_CE_Res	18.182	40.000	36.364	63.636	14.342	2.273
3_2021_2022	PM_10	Random	GBLUP_TRN	9.091	40.909	45.455	54.545	15.599	0.000
3_2021_2022	PM_20	IBD	GBLUP	22.727	47.727	50.000	63.636	10.978	21.905
3_2021_2022	PM_20	IBD	GBLUP_CE	22.727	45.909	45.455	59.091	10.595	26.733
3_2021_2022	PM_20	IBD	GBLUP_CE_Abs	22.727	44.091	45.455	54.545	9.352	31.959
3_2021_2022	PM_20	IBD	GBLUP_CE_mean	27.273	45.000	45.455	63.636	10.376	29.293
3_2021_2022	PM_20	IBD	GBLUP_CE_Res	31.818	48.182	47.727	72.727	11.579	20.755
3_2021_2022	PM_20	IBD	GBLUP_TRN	40.909	58.182	59.091	77.273	10.884	0.000
3_2021_2022	PM_20	Random	GBLUP	40.909	54.091	54.545	68.182	8.951	−1.681
3_2021_2022	PM_20	Random	GBLUP_CE	36.364	50.909	50.000	68.182	9.535	4.464
3_2021_2022	PM_20	Random	GBLUP_CE_Abs	40.909	52.273	50.000	68.182	8.368	1.739
3_2021_2022	PM_20	Random	GBLUP_CE_mean	40.909	52.727	50.000	68.182	9.141	0.862
3_2021_2022	PM_20	Random	GBLUP_CE_Res	40.909	54.091	54.545	68.182	8.145	−1.681
3_2021_2022	PM_20	Random	GBLUP_TRN	40.909	53.182	50.000	68.182	9.104	0.000
